# Deep learning neural network image analysis of immunohistochemical protein expression reveals a significantly reduced expression of biglycan in breast cancer

**DOI:** 10.1371/journal.pone.0282176

**Published:** 2023-03-27

**Authors:** Ana Paula Thiesen, Bruna Mielczarski, Ricardo Francalacci Savaris

**Affiliations:** 1 Universidade Federal do Rio Grande do Sul, Postgraduate Program in Health Science: Surgical Sciences, Porto Alegre, Rio Grande do Sul, Brazil; 2 Department of Obstetrics and Gynecology, Universidade Federal do Rio Grande do Sul, Porto Alegre, Rio Grande do Sul, Brazil; Universidade Federal do Rio de Janeiro, Instituto de Bioquimica Medica, BRAZIL

## Abstract

New breast cancer biomarkers have been sought for better tumor characterization and treatment. Among these putative markers, there is Biglycan (BGN). BGN is a class I small leucine-rich proteoglycan family of proteins characterized by a protein core with leucine-rich repeats. The objective of this study is to compare the protein expression of BGN in breast tissue with and without cancer, using immunohistochemical technique associated with digital histological score (D-HScore) and supervised deep learning neural networks (SDLNN). In this case-control study, 24 formalin–fixed, paraffin-embedded tissues were obtained for analysis. Normal (n = 9) and cancerous (n = 15) tissue sections were analyzed by immunohistochemistry using BGN monoclonal antibody (M01-Abnova) and 3,3’-Diaminobenzidine (DAB) as the chromogen. Photomicrographs of the slides were analysed with D-HScore, using arbitrary DAB units. Another set (n = 129) with higher magnification without ROI selection, was submitted to the inceptionV3 deep neural network image embedding recognition model. Next, supervised neural network analysis, using stratified 20 fold cross validation, with 200 hidden layers, ReLu activation, and regularization at α = 0.0001 were applied for SDLNN. The sample size was calculated for a minimum of 7 cases and 7 controls, having a power = 90%, an α error = 5%, and a standard deviation of 20, to identify a decrease from the average of 40 DAB units (control) to 4 DAB units in cancer. BGN expression in DAB units [median (range)] was 6.2 (0.8 to 12.4) and 27.31 (5.3 to 81.7) in cancer and normal breast tissue, respectively, using D-HScore (p = 0.0017, Mann-Whitney test). SDLNN classification accuracy was 85.3% (110 out of 129; 95%CI = 78.1% to 90.3%). BGN protein expression is reduced in breast cancer tissue, compared to normal tissue.

## Introduction

In women, breast cancer is the most commonly diagnosed cancer in the world and the leading cause of death from cancer [1]. Breast cancers are heterogeneous in nature, both at the histological and molecular levels, and the molecular profiling of breast cancer guides diagnostic and therapeutic strategies for the disease [2]. New breast cancer biomarkers have been sought in order to better characterize tumors and to select the best possible treatment [3]. Small leucine-rich proteoglycans (SLRPs), a diverse sub-group of proteoglycans, are involved in matrix organization and the regulation of cell growth and signaling [4]. Biglycan (BGN) is one SLRP whose gene has been mapped to the Xq28 chromosome [5]. BGN is a class I SLRP characterized by a protein core with leucine-rich repeats and is composed of 331 amino acids and a molecular weight of 42 kDa [6]. The molecular weight of BGN increases up to 100–250 kDa, when fully glycosylated. This glycosylation is due to the presence of two chondroitin/dermatan sulfate and glycosaminoglycan chains covalently attached to the N-terminal region [7]. The glycosaminoglycan chains consist of repeating disaccharide units of either chondroitin sulfate or dermatan sulfate and are attached to the core protein via an O-linked glycosidic bond. This proteoglycan is ubiquitously expressed; it can be incorporated into the extracellular matrix (ECM) or exist in the blood in its soluble form under certain disease conditions [8, 9].

BGN could alter tumor proliferation by modulating the receptors and cellular expression molecules within the tumor microenvironment [9]. Zhao *et al*., using a cancer microarray database and a web-based data-mining platform (Oncomine), have reported that BGN gene expression was upregulated in breast and other cancers [10]. However, the clinical impact of BGN on cancer is still poorly understood and sometimes contradictory. For instance, in bladder cancer, silencing of BGN resulted in enhanced tumor cell proliferation, indicating that BGN acts as a growth suppressor in this disease [11], while another study using animal model found that the inhibition of stromal BGN promoted normalization of the tumor microenvironment and enhanced chemotherapeutic efficacy in mice injected with breast cancer cells [12]. Bischof *et al*. demonstrated that the injection of normal, early stage, embryonic mesenchyme cells was sufficient to induce differentiation and suppress growth of mouse mammary tumor epithelial cells both *in vitro* and *in vivo*; they reported that BGN was required for tumor normalization [13].

These apparent contradictions may be explained by the different types of models used, i.e., *in vitro*, animal model, and detection method (mRNA, or immunohistochemistry). The type of antibodies used in immunohistochemistry and the specific site for BGN identification may also explain some of these discrepancies. For instance, mature and functionally active BGN protein was detected using a polyclonal antibody after glycosaminoglycan removal by enzymatic digestion with chondroitinase ABC [14]. This selective removal was performed, because the presence of a complex of two chondroitin/dermatan sulfates with glycosaminoglycan chains could hinder antibody binding, leading to the misinterpretation of results [14]. Nevertheless, this polyclonal antibody was discontinued, making new studies with this antibody unsuitable. These differences are more evident when comparisons between different antibodies are applied in the same tissue. For instance, the Human Protein Atlas database [15] has two antibodies validated against BGN to be used in immunohistochemistry: HPA003157 (Sigma, Aldrich), a polyclonal antibody that targets 140 amino acids, and H00000633-M01 (Abnova, Taipei, Taiwan), and a monoclonal antibody against the full sequence of BGN (368 amino acids); the protein expression of BGN in breast cancer is different between these two antibodies. While the former had 8.3% negative expression (1 out 12 cases), the latter had a negative expression in 75% (9 out 12 cases) [16]. In addition, manual evaluation of immunohistochemically stained specimens is a subjective and highly individual task, which, as has been reported by others, depends on intra- and inter-observer variability [17].

To reduce the highly subjective and easily biased nature of these tasks, the digital histological scoring method (D-HSCORE) has been reported [18]. Another emerging area in image analysis is deep learning. Deep learning (DL) is a form of machine learning that relies on both supervised and unsupervised learning; DL applied to digital pathology uses artificial neural networks (ANN) to determine if the output or interpretation of a digital image is correct [19]. ANN uses multiple layers of calculations imitating the complex network of neurons in the human brain to analyze this complex data [19].

Data on BGN expression in human breast cancer is scant and contradictory [13, 20], and little information is available for human BGN protein expression *in vivo* using an antibody against the full length of the protein. Therefore, the objective of this study is to verify the immunohistochemical expression of BGN in breast cancer biopsies compared to normal breast tissue using a validated monoclonal antibody and two digital imaging methods of analysis: D-HSCORE and deep learning neural network image analysis.

## Material and methods

### Ethics statement

This study was submitted and approved by Hospital de Clínicas de Porto Alegre Ethical Review Board, under the approval number 2019/0337 and registered at *Plataforma Brasil* under the certificate of submission for ethical analysis (CAAE 15329119.9.0000.5327).

### Study design and setting

In this case-control study, paraffin blocks were obtained from the pathological archive of Hospital de Clínicas in Porto Alegre, Brazil. Slides were dated between January 1st, 2012, and December 30th, 2015. The original pathological report was reviewed by a certified board pathologist to confirm the diagnosis of benign and cancerous breast tissue. The study was conducted between May 20, 2019, and July 30, 2020.

### Patients and methods

Women with diagnoses of invasive ductal carcinoma and those who underwent breast surgery for benign conditions (e.g., mammoplasty, benign mammary cyst) were included in the sample. Patients with lobular carcinoma or intraductal papilloma who had undergone chemotherapy or radiotherapy and aged below 20- and over 79-years-old were excluded. These cases were excluded since chemo and radiotherapy may change protein expression of the tumor.

### Variables

BGN protein expression was the primary continuous variable, i.e., DAB units, varying between 0 and 255 units.

Healthy breast tissue (benign—control group) and breast cancer tissue (cancer) were categorical data. Other variables were age and ethnicity. Estrogen and progesterone receptor status were described in cancerous tissues, along with human epidermal growth factor receptor 2 (HER2), Nottingham Grading and tumor staging.

### Data sources / measurement

#### Immunohistochemistry

Immunohistochemistry methodology was performed according to standard technique [21] and as previously reported by our group with minor modifications [22]. Modifications included deparaffinized at 75°C for two hours, followed by xylol rinse, rehydrated in successive steps of ethanol, water, and phosphate-buffered saline solution (PBS); slide incubation was done in sodium citrate solution, pH 6 at 90°C for 45 min. for antigen retrieval; primary antibody against the full length of recombinant BGN was BGN monoclonal antibody (M01), clone 4E1-1G7, IgG2a kappa (Abnova, Taipei, Taiwan). It was used at dilution 1:1000 at pH 6 and incubated overnight. After 2 x 5 minutes in a PBS rinse, secondary antibody anti-mouse IgG (whole molecule), namely peroxidase antibody produced in rabbit (A9044, Sigma-Aldrich, Darmstadt, Germany), was incubated for 90 minutes at 22°C in the same chamber using a dilution of 1:1000. The primary antibody visualization and counterstained were performed as previously reported [22]. Negative controls were obtained by replacing the primary antibody with mouse IgG2a, kappa monoclonal [18C8BC7AD10]—Isotype Control (ab170191)—Abcam, Cambridge, UK). Human lung cancer samples were used as external positive control. These procedures followed the REporting recommendations for tumor MARKer prognostic studies (REMARK) guidelines [23].

Images from stained sections were obtained using an optical microscope (Olympus BX51 microscope; Olympus Optical Co., Tokyo, Japan) with a 40x objective U Plan Fluorite dry objective (numerical aperture 0.65 mm, Olympus). A digital color camera (Olympus DP73; OM Digital Solutions Co., Tokyo, Japan) captured digital images, at a size of 4800 x 3600 pixels (resolution: 1 mm = 6000 pixels), under standard conditions for ImageJ analysis. Another set of images (n = 129 photomicrographs) with a 100x objective (UPLFL 100x; Oil Immersion, Olympus) were taken of entire slides for supervised deep learning neural network analysis.

### Image analysis with ImageJ

Photomicrographs were coded and blindly analyzed using Digital HSCORE (D-HSCORE) as previously reported [18, 24, 25]. Briefly, only the glandular and tumor sites of the tissue sections were selected as regions of interest (ROI). After selecting the ROI, images were submitted for “color deconvolution” analysis. The image with DAB staining was used for analysis.

### Supervised deep learning neural network

The 129 photomicrographs of DAB-only images, with 100x magnification and without ROI selection, were submitted to the inceptionV3 deep neural network image embedding recognition model using Orange 3.31.0 software (University of Ljubljana, Slovenia). Next, supervised neural network analysis (SDLNN), using stratified 20-fold cross validation with 200 hidden layers, ReLu activation, and regularization at α = 0.0001, were submitted to SDLNN, in Orange software.

### Bias

Bias was reduced by using D-HSCORE and SDLNN.

### Study size

The sample size for ImageJ analysis was calculated according to the literature [26] in order to have a power = 90%, an α error = 5%, and a standard deviation of 20, to identify a decrease from the average of 40 DAB units (control) to 4 DAB units in cancer. With these figures, at least 7 samples in each group were necessary.

Sample size for supervised neural network analysis was chosen for convenience after obtaining the maximum number of photomicrographs from the slides.

### Quantitative variables

The average DAB units intensity, derived from up to three images obtained from color deconvolution, was calculated according to the formula: ƒ = 255-i, where ƒ = final DAB intensity, and i = mean DAB intensity obtained from the software, as previously described [18].

### Statistical analysis

Groups, with categorical data, were compared using Fisher’s exact test. Continuous data of BGN expression, in arbitrary DAB units, between groups, were compared using unpaired Student *t*-test with Welch’s correction if data had a Gaussian distribution and different SDs, otherwise Mann-Whitney test was used. D’Agostino & Pearson omnibus normality test was used to verify Gaussian distribution. These analyses were performed using GraphPad Prism version 9.3.1 for Macintosh (GraphPad Software Inc. San Diego, CA).

Deep neural network image embedding recognition model analysis was performed using Orange 3.31.0 software (University of Ljubljana, Slovenia). Supervised neural network was set with 200 hidden layers, the rectified linear activation function (ReLu) was used for activation, Adam was used as the optimization algorithm [27], regularization was set at α = 0.0001, and maximum number of iterations was 300, with training replication. Test and training were set using a stratified 20-fold cross validation.

## Results

### Participants and descriptive data

A total of 24 samples were obtained for the study: benign = 9; cancerous = 15. The mean age between groups was not significant (p = 0.1; unpaired Student *t*-test). Details of the sampled population are depicted in Table 1.

**Table 1:** Characteristics of the studied population.

Characteristics	Breast Cancer (n = 15)	Controls (n = 9)	*P*
Age (years) mean (SD)	51.6(11.6)	43.2(12.2)	0.1^a^
Ethnicity			
caucasian/non-caucasian	14/1	8/1	1^b^
**Pathology Report (n)**			
Invasive ductal carcinoma	15		
Normal breast tissue		2	
normal with adenosis		2	
stromal fibrosis		4	
Fibro-microcystic changes		1	
Estrogen Receptor (positive/negative)	6/9		
Progesterone Receptor (positive/negative)	10/5		
HER2 (n)%			
0, +1	11 (73.4)		
+2	2 (13.3)		
+3	2 (13.3)		
Nottingham Grading (n)%			
I	1 (6.7)		
II	8 (53.3)		
III	6 (40)		
Staging—n (%)			
I	3 (20)		
IIA	5 (33.3)		
IIB	1 (6.7)		
IIIA	4 (26.7)		
IIIB	1 (6.7)		
IV	1 (6.7)		

^a^ Unpaired Student *t*-test with Welch’s correction

^b^ Fisher’s exact test

### Outcome data

**Fig 1:**
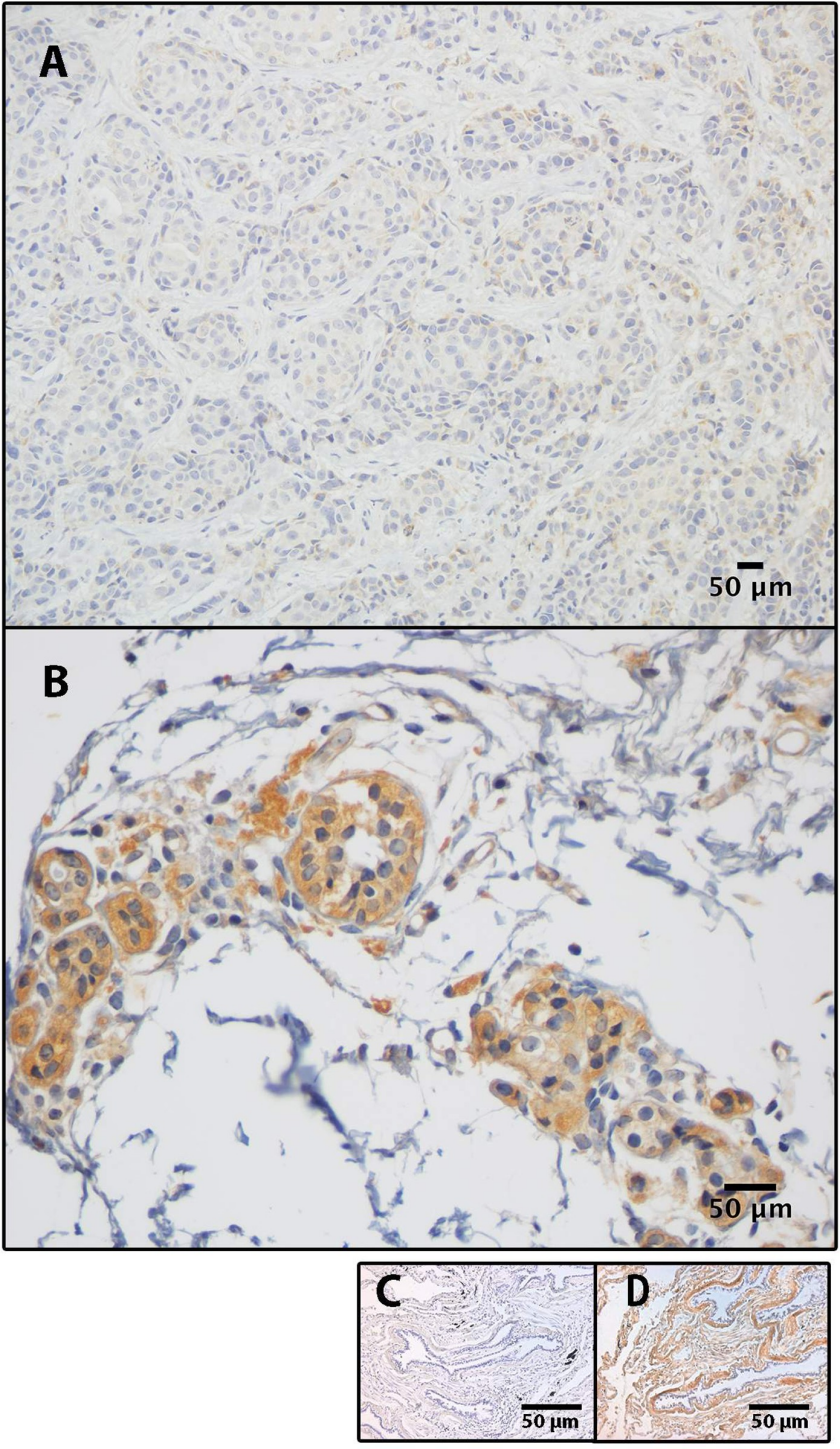
Photomicrographs of BGN protein expression in (A) invasive ductal carcinoma, and in (B) normal breast tissue. Lung tissue was used as an external negative (C) and positive (D) control. Bars represent 50 μm. Scatter dot plot (E) demonstrates a significant reduction of BGN protein expression in breast cancer tissue, compared to benign breast tissue (p = 0.0017; Mann-Whitney test). Each dot represents a sample, bars represent median values.

BGN protein expression in DAB units [median (range)] was significantly lower in breast tissue with cancer 6.2 (0.8 to 12.4) compared to benign breast tissue 27.31(5.3 to 81.7) as shown in Fig 1 (p = 0.0017, Mann-Whitney test).

The area [median(range) in mm²] analyzed between benign tissue [0.67(0.39 to 4.6)] and cancer tissue [1.1(0.08 to 2.8)] was not significantly different (p = 0.48; Mann-Whitney U test).

### Supervised neural network analysis

**Fig 2:**
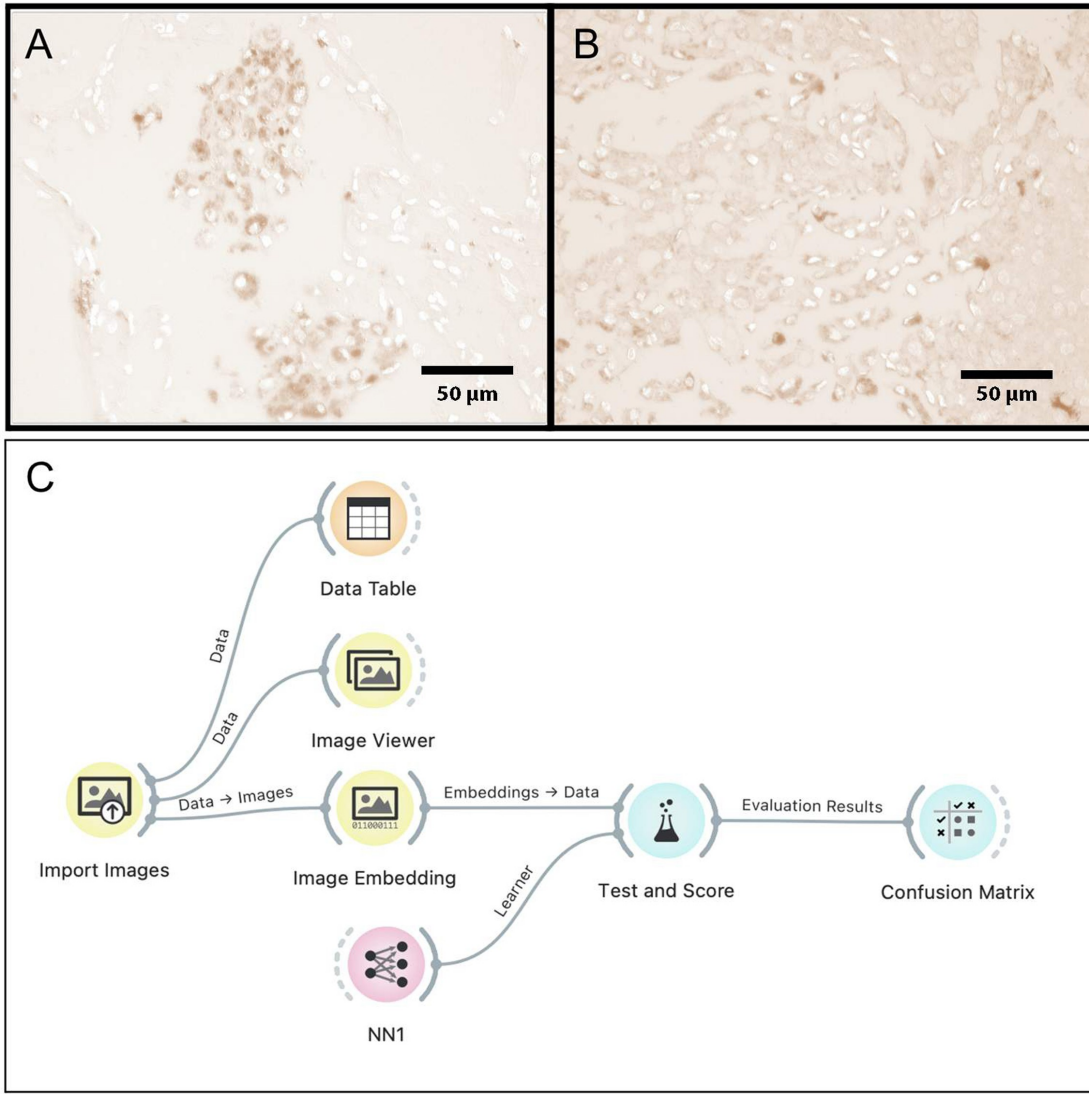
Example of BGN expression in DAB only in photomicrographs, after color deconvolution in normal breast tissue (A) and with cancer (B), bars represent 50 μm. Flowchart of the supervised neural network analysis using Orange 3 software (C). NN1:supervised neural network analysis.

A total of 129 high-power magnification (100x) photomicrographs with DAB staining only and derived from ImageJ (benign, n = 69; cancer, n = 60) were submitted for supervised neural network analysis (Fig 2).

The performance of the supervised neural network analysis yielded an area under the curve of 94.3%, further details are depicted in Table 2.

**Table 2:** Performance of supervised neural network analysis for classifying samples as benign or cancerous based on DAB expression only.

confusion matrix				Parameter	Results (95% confidence interval)	
		Predicted		Accuracy	85.3	(78.1–90.3)
		benign	Cancer	F1	86.1	
actual	benign	59	10	Precision	86.8	(76.7–92.8)
	cancer	9	51	Recall	85.5	(75.3–91.9)

F1: weighted harmonic mean of precision and recall

Precision: the proportion of true positives among instances classified as positive

Recall: the proportion of true positives among all positive instances in the data)

## Discussion

BCG expression, using the antibody M01-4E1-1G7 under the methodological conditions described here, was mainly located in the cytoplasm and the extracellular matrix. The location of the protein is in accordance with others. It has been reported by different authors that BGN protein expression in gastric cancer was mainly located in the cytoplasm of epithelial cells [28, 29].

In breast cancer tissues, the expression of BGN was significantly lower when compared to normal breast tissue. These results are in accordance with those published by Bischof *et al*. who reported that adding BGN to an *in vitro* model has the ability to reverse neoplastic progression and ‘reboot’ breast cancer cells [13]. Nevertheless, our results are different from those published by others [10, 12]. Possible explanations for these discrepancies can be related to a) number of cases, b) type of model used, c) type of antibody, and d) its biological effect. Apparently, in the human protein atlas, only two cases were accessed [16], while our sample had nine cases. The standard deviation of the BGN protein expression in normal tissue was high: two cases had low levels of expression (5.39 and 8 DAB units), while one case reached 81 DAB units (Fig 1). The use of different species may also explain these differences. Cong *et al*. noted that suppressing stromal BGN may yield a potent and superior anticancer effect in breast cancer induced in BGN knockout mice, compared to wild type [12]. While Cong *et al*. used a knockout mice model, we used human breast cancer tissue. In addition, data obtained from Zhao *et al*. was based on an Oncomine database using mRNA [10]. Another explanation for the lower levels of BGN protein expression in breast cancer tissue may be related to the full-length monoclonal antibody that was used here. Identifying various segments of the BGN protein may yield different outcomes due to the presence of chondroitin/dermatan sulfate + glycosaminoglycan side chains. The presence of these side chains may hinder antibody binding, leading to the misinterpretation of results [14]. Finally, the amount of BGN in tissue sections does not necessarily reflect its biological effect as it mainly indicates BGN that has been sequestered in the extracellular matrix, for example, as part of the fibrotic scar [30]. Despite these disagreements, the difference in the protein expression in benign and malignant breast tissue reveals that the local microenvironment, including the ECM, may have an important role in controlling cell growth, survival, and fate determination [20].

This study has some limitations. We neither analyzed subgroups, nor did any mechanistic experiment. In addition, we are unaware if there is a difference in the BGN expression across the menstrual cycle, which may explain the differences in BGN expression in normal breast tissue. Finally, we did not make a side-by-side comparison of different antibodies in order to identify putative differences.

The results here are strengthened by several aspects. The use of ImageJ software reduced the subjective bias of DAB quantification. The artificial intelligence was able to classify, with a high degree of accuracy, 129 photomicrographs based only on DAB expression, confirming the results obtained with ImageJ analysis. The use of both techniques together is not widely used yet, but it is promising. The criteria used for classification of the slides by the artificial intelligence was not completely understood; it is likely that the artificial intelligence used other factors beyond DAB expression. However, it is unlikely that the shape of the cells seen in DAB pictures had a major influence in the classification of the slides. The use of monoclonal antibody and non-specific primary antibody, as negative control, are evidence of the special care taken in the quality control of the immunohistochemical methodology. Immunohistochemical staining was performed using a non-specific primary antibody; this procedure has been considered a better methodology, compared to the omission of the primary antibody. Lung cancer was used as external positive and negative controls. External validity is expected once the same methodology is applied.

With our results, further studies may investigate the use of BGN as a biomarker or as a prognostic factor in breast cancer. The functional significance and the role of BGN alterations in breast tumorigenesis and progression remain to be determined.

## Supporting information

S1 File (XLSX)

## References

[1] Sung H, Ferlay J, Siegel RL, Laversanne M, Soerjomataram I, Jemal A, et al. Global Cancer Statistics 2020: GLOBOCAN Estimates of Incidence and Mortality Worldwide for 36 Cancers in 185 Countries. CA Cancer J Clin. 2021;71: 209–249. doi: 10.3322/caac.21660 33538338

[2] Zubair M, Wang S, Ali N. Advanced Approaches to Breast Cancer Classification and Diagnosis. Front Pharmacol. 2020;11: 632079. doi: 10.3389/fphar.2020.632079 33716731 PMC7952319

[3] Ramirez-Valles EG, Rodríguez-Pulido A, Barraza-Salas M, Martínez-Velis I, Meneses-Morales I, Ayala-García VM, et al. A Quest for New Cancer Diagnosis, Prognosis and Prediction Biomarkers and Their Use in Biosensors Development. Technol Cancer Res Treat. 2020;19: 1533033820957033. doi: 10.1177/1533033820957033 33107395 PMC7607814

[4] Appunni S, Anand V, Khandelwal M, Gupta N, Rubens M, Sharma A. Small Leucine Rich Proteoglycans (decorin, biglycan and lumican) in cancer. Clin Chim Acta. 2019;491: 1–7. doi: 10.1016/j.cca.2019.01.003 30629950

[5] McBride OW, Fisher LW, Young MF. Localization of PGI (biglycan, BGN) and PGII (decorin, DCN, PG-40) genes on human chromosomes Xq13-qter and 12q, respectively. Genomics. 1990;6: 219–225. doi: 10.1016/0888-7543(90)90560-h 1968422

[6] Bianco P, Fisher LW, Young MF, Termine JD, Robey PG. Expression and localization of the two small proteoglycans biglycan and decorin in developing human skeletal and non-skeletal tissues. J Histochem Cytochem. 1990;38: 1549–1563. doi: 10.1177/38.11.2212616 2212616

[7] Fisher LW, Termine JD, Young MF. Deduced protein sequence of bone small proteoglycan I (biglycan) shows homology with proteoglycan II (decorin) and several nonconnective tissue proteins in a variety of species. J Biol Chem. 1989;264: 4571–4576. 2647739

[8] Tufvesson E, Malmström J, Marko-Varga G, Westergren-Thorsson G. Biglycan isoforms with differences in polysaccharide substitution and core protein in human lung fibroblasts. Eur J Biochem. 2002;269: 3688–3696. doi: 10.1046/j.1432-1033.2002.03058.x 12153565

[9] Nastase MV, Young MF, Schaefer L. Biglycan: a multivalent proteoglycan providing structure and signals. J Histochem Cytochem. 2012;60: 963–975. doi: 10.1369/0022155412456380 22821552 PMC3527886

[10] Zhao S-F, Yin X-J, Zhao W-J, Liu L-C, Wang Z-P. Biglycan as a potential diagnostic and prognostic biomarker in multiple human cancers. Oncol Lett. 2020;19: 1673–1682. doi: 10.3892/ol.2020.11266 32194659 PMC7039163

[11] Niedworok C, Röck K, Kretschmer I, Freudenberger T, Nagy N, Szarvas T, et al. Inhibitory role of the small leucine-rich proteoglycan biglycan in bladder cancer. PLoS One. 2013;8: e80084. doi: 10.1371/journal.pone.0080084 24223213 PMC3819308

[12] Cong L, Maishi N, Annan DA, Young MF, Morimoto H, Morimoto M, et al. Inhibition of stromal biglycan promotes normalization of the tumor microenvironment and enhances chemotherapeutic efficacy. Breast Cancer Res. 2021;23: 51. doi: 10.1186/s13058-021-01423-w 33966638 PMC8108358

[13] Bischof AG, Yüksel D, Mammoto T, Mammoto A, Krause S, Ingber DE. Breast cancer normalization induced by embryonic mesenchyme is mediated by extracellular matrix biglycan. Integr Biol. 2013;5: 1045–1056. doi: 10.1039/c3ib40103k 23817524

[14] Hara T, Yoshida E, Shinkai Y, Yamamoto C, Fujiwara Y, Kumagai Y, et al. Biglycan Intensifies ALK5-Smad2/3 Signaling by TGF-β and Downregulates Syndecan-4 in Cultured Vascular Endothelial Cells. J Cell Biochem. 2017;118: 1087–1096. 27585241 10.1002/jcb.25721 PMC6221004

[15] Uhlén M, Fagerberg L, Hallström BM, Lindskog C, Oksvold P, Mardinoglu A, et al. Proteomics. Tissue-based map of the human proteome. Science. 2015;347: 1260419. doi: 10.1126/science.1260419 25613900

[16] Expression of BGN in cancer—Summary—The Human Protein Atlas. [cited 6 Jan 2022]. Available: https://www.proteinatlas.org/ENSG00000182492-BGN/pathology

[17] Rüdiger T, Höfler H, Kreipe H-H, Nizze H, Pfeifer U, Stein H, et al. Quality assurance in immunohistochemistry: results of an interlaboratory trial involving 172 pathologists. Am J Surg Pathol. 2002;26: 873–882. doi: 10.1097/00000478-200207000-00005 12131154

[18] Fuhrich DG, Lessey BA, Savaris RF. Comparison of HSCORE assessment of endometrial beta3 integrin subunit expression with digital HSCORE using computerized image analysis (ImageJ). Anal Quant Cytopathol Histpathol. 2013;35: 210–216. 24341124 PMC4090774

[19] Aeffner F, Zarella MD, Buchbinder N, Bui MM, Goodman MR, Hartman DJ, et al. Introduction to Digital Image Analysis in Whole-slide Imaging: A White Paper from the Digital Pathology Association. J Pathol Inform. 2019;10: 9. doi: 10.4103/jpi.jpi_82_18 30984469 PMC6437786

[20] Bruno RD, Smith GH. A potential mechanism for extracellular matrix induction of breast cancer cell normality. Breast cancer research: BCR. 2014. p. 302. doi: 10.1186/bcr3617 25927296 PMC3978454

[21] Rasmussen OF, Rudbeck L. Immunohistochemistry: A Dako Perspective. Handbook of Practical Immunohistochemistry. 2015. pp. 57–67.

[22] Lopes Q dos A, dos Anjos Lopes Q, de Paula Guedes neto E, Gross LA, Pedrini JL, Savaris RF. Heart and Neural Crest Derivatives Expressed Transcript 2 (HAND2) is Reduced in Women with Breast Cancer. A Case-Control Study. Clinical Oncology and Research. 2019. pp. 1–6. doi: 10.31487/j.cor.2019.5.12

[23] Altman DG, McShane LM, Sauerbrei W, Taube SE. Reporting Recommendations for Tumor Marker Prognostic Studies (REMARK): explanation and elaboration. PLoS Med. 2012;9: e1001216. doi: 10.1371/journal.pmed.1001216 22675273 PMC3362085

[24] Ruifrok AC, Johnston DA. Quantification of histochemical staining by color deconvolution. Anal Quant Cytol Histol. 2001;23: 291–299. 11531144

[25] Grudzinski M, Fuhrich DG, Savaris RF. Expression of elafin in fallopian tubes of ectopic pregnancies is reduced. Appl Immunohistochem Mol Morphol. 2015;23: 349–354. doi: 10.1097/PAI.0000000000000091 25356943

[26] Julious SA. Sample Sizes for Clinical Trials. CRC Press; 2009.

[27] Kingma DP, Ba JL. ADAM: A METHOD FOR STOCHASTIC OPTIMIZATION. In: Bengio Y LY, editor. 3rd International Conference on Learning Representations. 7–9 May 2015. pp. 1–15.

[28] Hu L, Duan Y-T, Li J-F, Su L-P, Yan M, Zhu Z-G, et al. Biglycan enhances gastric cancer invasion by activating FAK signaling pathway. Oncotarget. 2014;5: 1885–1896. doi: 10.18632/oncotarget.1871 24681892 PMC4039113

[29] Pinto F, Santos-Ferreira L, Pinto MT, Gomes C, Reis CA. The Extracellular Small Leucine-Rich Proteoglycan Biglycan Is a Key Player in Gastric Cancer Aggressiveness. Cancers. 2021;13. doi: 10.3390/cancers13061330 33809543 PMC8001774

[30] Schaefer L. Small Leucine-Rich Proteoglycans in Kidney Disease. Journal of the American Society of Nephrology. 2011. pp. 1200–1207. doi: 10.1681/ASN.2010050570 21719787

